# Breed and adaptive response modulate bovine peripheral blood cells’ transcriptome

**DOI:** 10.1186/s40104-017-0143-y

**Published:** 2017-01-25

**Authors:** Nataliya Pošćić, Tommaso Montanari, Mariasilvia D’Andrea, Danilo Licastro, Fabio Pilla, Paolo Ajmone-Marsan, Andrea Minuti, Sandy Sgorlon

**Affiliations:** 1 Department of Agriculture, Food, Environment and Animal Science (DI4A), University of Udine, via delle Scienze 206, 33100 Udine, Italy; 2 Department of Agricultural, Environmental and Food Sciences, University of Molise, via F. De Sanctis snc, 86100 Campobasso, Italy; 3 CBM S.c.r.l, SS 14 – km 163.5 AREA Science Park, 34149 Basovizza, TS Italy; 4 Institute of Zootechnics, Catholic University of the Sacred Heart, via Emilia Parmense 84, 29133 Piacenza, Italy

**Keywords:** Acute phase proteins, Adaptive response, Dynamic impact approach (DIA), Hypothalamic-pituitary-adrenal (HPA) axis, RNA-Seq, Stress response, Transcriptomics

## Abstract

**Background:**

Adaptive response includes a variety of physiological modifications to face changes in external or internal conditions and adapt to a new situation. The acute phase proteins (APPs) are reactants synthesized against environmental stimuli like stress, infection, inflammation.

**Methods:**

To delineate the differences in molecular constituents of adaptive response to the environment we performed the whole-blood transcriptome analysis in Italian Holstein (IH) and Italian Simmental (IS) breeds. For this, 663 IH and IS cows from six commercial farms were clustered according to the blood level of APPs. Ten extreme individuals (five APP+ and APP- variants) from each farm were selected for the RNA-seq using the Illumina sequencing technology. Differentially expressed (DE) genes were analyzed using dynamic impact approach (DIA) and DAVID annotation clustering. Milk production data were statistically elaborated to assess the association of APP+ and APP- gene expression patterns with variations in milk parameters.

**Results:**

The overall de novo assembly of cDNA sequence data generated 13,665 genes expressed in bovine blood cells. Comparative genomic analysis revealed 1,152 DE genes in the comparison of all APP+ vs. all APP- variants; 531 and 217 DE genes specific for IH and IS comparison respectively. In all comparisons overexpressed genes were more represented than underexpressed ones. DAVID analysis revealed 369 DE genes across breeds, 173 and 73 DE genes in IH and IS comparison respectively. Among the most impacted pathways for both breeds were vitamin B6 metabolism, folate biosynthesis, nitrogen metabolism and linoleic acid metabolism.

**Conclusions:**

Both DIA and DAVID approaches produced a high number of significantly impacted genes and pathways with a narrow connection to adaptive response in cows with high level of blood APPs. A similar variation in gene expression and impacted pathways between APP+ and APP- variants was found between two studied breeds. Such similarity was also confirmed by annotation clustering of the DE genes. However, IH breed showed higher and more differentiated impacts compared to IS breed and such particular features in the IH adaptive response could be explained by its higher metabolic activity. Variations of milk production data were significantly associated with APP+ and APP- gene expression patterns.

**Electronic supplementary material:**

The online version of this article (doi:10.1186/s40104-017-0143-y) contains supplementary material, which is available to authorized users.

## Background

In the context of adaptation, stress response is an important neurobehavioral and physiological reaction and it is essential for the survival of living organisms. In response to a stressor, the body orchestrates changes in brain activity followed by the secretion of “stress mediators”, including cytokines, metabolic hormones and corticosteroids [1].

The body’s response during the first stage of stress is known as fight-or-flight response. It includes the activation of sympathetic nervous system and the stimulation of the production of adrenaline and noradrenaline by adrenal glands. These molecules increase the heart rate and the glycemia and modify blood distribution to supply greater levels of glucose to organs where they are needed, like brain and skeletal muscles. Shortly after, the hypothalamic-pituitary-adrenal (HPA) axis is activated and releases corticosteroids (in particular adrenal glucocorticoids). In turn, these produce a negative feedback onto immune cells and suppress further synthesis and release of cytokines, thereby protecting the host from the detrimental consequences of an overactive immune response (e.g., tissue damage, autoimmunity, septic shock) [2].

The long-term activation of the stress-response mechanisms may also cause irreversible damages, like cardiovascular diseases, immunosuppression, dysfunction of digestive and reproductive systems, type-II diabetes mellitus, impairment of thyroid function, weakening and loss of body lean mass [3, 4]. Such pre-pathological or pathological consequences seriously affect not only the efficiency of animal production and the quality of the product, but undoubtedly reduce animal welfare.

During the acute phase reaction (APR), the body mounts a multifactorial response trying to remove or replace damaged tissues and one of the mechanisms involved is the secretion of the so-called acute phase proteins (APPs). The concentration of some APPs increases several fold during the APR, while others, including albumin, decreases as the liver switches the production of proteins towards the synthesis of proteins required to deal with the damage [5, 6].

In ruminants, APPs are very sensitive factors that allow the early and precise detection of inflammation [7]. The most frequently investigated proteins in cattle are: haptoglobin (Hp), serum amyloid A (SAA), fibrinogen (Fb), ceruloplasmin, α 1-antitrypsin and α 1-acid glycoprotein (α1-AGP) [5, 8–10]. It is possible that the synthesis of APPs in cattle is influenced by cortisol [11, 12], which is the key effector molecule of the HPA axis and is recognized as the physiological response to stress [13–16].

Stress response mechanisms in cattle are still not well understood and the research is complicated by individual differences in stress response [17]. Today, the investigation of how dairy cattle adapt to intensive production is particularly important, since the animal welfare is a growing public concern and stressed animals are less efficient, producing less than predicted by their genetic potential mostly due to a higher environmental impact.

Next-generation high-throughput RNA sequencing technology (RNA-seq) is a recently-developed method for discovering, profiling, and quantifying RNA transcripts. Such approach is used to analyze the continually changing cellular transcriptome and might help identifying gene patterns involved in adaptive response. Applicability of RNA-seq for transcriptome analysis of whole blood samples was already confirmed by many research groups [18–20]. Among the most distinct advantages of RNA-seq over prior methods for mapping and quantifying the transcriptome are unbiased whole-transcriptome profiling, higher sensitivity and accurate estimation of lowly expressed transcripts in peripheral whole blood with or without globin depletion [20].

Up to date RNA-seq technique was highly applied for the assessment of changes in blood transcript abundance in response to stress events, pathogenic processes, and specific physiological and metabolic statuses in dairy cattle [19–22]. However, no study has comprehensively evaluated the adaptive response on molecular changes in dairy cattle whole blood cell transcriptome as an indicator of immune activity without the visible environmental perturbations.

In this context, we used a whole-transcriptome analysis to understand if and how differential gene expression contributes to such a complex phenomenon as adaptive response. Therefore, in the present research, the transcriptome of blood cells was analyzed in selected bovines belonging to Italian Holstein (IH) and Italian Simmental (IS) breeds from six commercial farms in Friuli-Venezia Giulia region, Italy. Cows were clustered for blood APPs, plasma Zn, milk cortisol and somatic cell count (SCC) in milk. The analysis included RNA isolation from blood [23], sequencing by RNA-seq with Illumina pipeline [24–27] and the use of the normalized data for the identification of genes expression of which is significantly associated to the adaptive response to the undefined stress conditions. Genes and metabolic pathways were further analyzed using the dynamic impact approach (DIA) and DAVID online software tool [28].

## Methods

### Animals and management

A total of 663 IH and IS cows from six commercial farms in Friuli-Venezia Giulia region of Italy were included in the experiment. All animals were kept under the same feeding and management conditions and were in the stage of lactation. Farm veterinary practitioner confirmed that all sampled animals passed the preliminary veterinary checkup, were clinically healthy and were not under any treatments for at least 1 month before the collection day. Composition of herds and characteristics of animals included in the analysis are reported in the Tables 1 and 2.

**Table 1:** Composition of herds and number of animals included in the analysis

	F1	F2	F3	F4	F5	F6
Breed	IH	IH	IH	IS	IS	IS
Herd size	654	456	442	538	201	270
Dairy animals	347	250	235	280	119	147
First calving	131	85	82	86	41	36
Lactating cows	313	227	195	225	96	123
Cows >50 DIM	279	204	147	185	84	111
Sampled cows	184	112	75	126	78	88
of which:						
1^st^ parity	85	40	36	42	35	23
2^nd^ parity	49	33	18	31	11	19
3^rd^ parity	23	18	11	29	18	13
4^th^ parity	17	10	4	14	9	17
>4^th^ parity	10	11	6	10	5	16

(*F* farm, *IH* Italian Holstein, *IS* Italian Simmental, *DIM* days in milk)

**Table 2:** Characteristics of the sampled animals within each farm

	F1	F2	F3	F4	F5	F6
BCS	2.4 ± 0.4	2.4 ± 0.5	2.2 ± 0.5	3.0 ± 0.5	3.4 ± 0.4	2.8 ± 0.3
DIM	179 ± 63	179 ± 53	111 ± 62	160 ± 63	162 ± 99	166 ± 66
Milk yield, kg	42.9 ± 9.1	36.9 ± 7.7	33.7 ± 7.7	26.9 ± 7.8	30.2 ± 6.9	27.8 ± 1.5
Milk fat, %	3.3 ± 0.5	4.1 ± 0.6	3.5 ± 0.6	3.7 ± 0.7	4.1 ± 4.5	3.5 ± 0.7
Milk protein, %	3.1 ± 0.3	3.4 ± 0.3	3.1 ± 0.3	3.7 ± 0.3	3.6 ± 0.4	3.5 ± 0.3
Milk casein, %	2.5 ± 0.2	2.6 ± 0.2	2.5 ± 0.2	2.9 ± 0.2	2.8 ± 0.3	2.8 ± 0.3
Milk urea, mg/dL	18.2 ± 3.4	19.4 ± 4.2	20.8 ± 3.4	21.5 ± 5.5	20.5 ± 4.1	20.9 ± 4.2
SCC	369 ± 732	322 ± 594	439 ± 847	659 ± 1,210	181 ± 503	485 ± 1,319
Milk cortisol, pg/mL	492 ± 335	586 ± 840	562 ± 314	636 ± 275	448 ± 174	481 ± 319
Blood parameters:						
Ceruloplasmin, μmol/L	2.8 ± 0.5	2.9 ± 0.9	2.8 ± 0.6	3.2 ± 0.6	2.4 ± 0.7	2.4 ± 0.5
Total proteins, g/L	77.5 ± 7.8	80.0 ± 6.9	80.6 ± 7.2	81.0 ± 5.2	75.6 ± 5.1	79.7 ± 4.8
Albumin, g/L	37.3 ± 3.1	38.2 ± 3.5	35.3 ± 3.1	37.1 ± 2.1	36.7 ± 2.2	38.6 ± 1.6
Haptoglobin, g/L	0.40 ± 0.33	0.42 ± 0.47	0.50 ± 0.46	0.29 ± 0.30	0.33 ± 0.21	0.42 ± 0.42
Paraoxonase, U/mL	112 ± 26	100 ± 24	104 ± 25	88 ± 21	101 ± 16	90 ± 22
Zinc, μmol/L	14.8 ± 4.3	13.5 ± 2.5	12.1 ± 2.5	12.3 ± 2.7	13.0 ± 2.3	12.1 ± 1.8

(*F* farm, *BCS* body condition score, *DIM* days in milk, *SCC* somatic cell count, values are expressed as mean ± SD)

Farmers and farm veterinary practitioners gave an oral informed consent to the study and had a copy of all the data obtained from the laboratory analyses. All farms involved in the present study adhere to a high standard of veterinary care based on best practice manual under the supervision of the official veterinary service. Sample collection was approved by the Bioethics Committee of the University of Udine.

Cows were housed in a free stall barn with cubicle design and automated milking parlour. They were milked twice a day, at approximately 12 h interval. Cows had free access to water and were fed ad libitum twice a day a total mixed ration (TMR) based on corn silage and formulated to cover nutrient requirements [29]. TMR was administered after each milking. To ensure that no dietary variations occurred during the time window of the study, the ration formulation and the offered amount were recorded using registrations of the TMR mixed feeder. In Table 3 are summarized composition, chemical properties and nutritional values of the diet in the six commercial farms.

**Table 3:** Diet composition (kg/d) in the selected commercial farms, chemical properties and nutritional values of the rations

Item	Farms					
	F1	F2	F3	F4	F5	F6
Lucerne	8.0	4.4	5.0	4.5	3.5	4.5
Pasture hay			1.5	1.5	3.3	
Wheat silage		2.2				
Corn silage	22.5	20.0	15.0	20.0	32.0	20.0
Ryegrass silage		8.0				5.0
Cotton seeds		1.2	1.0	1.0		
Corn meal	5.2	6.0	6.0	4.5		6.0
Soybean meal	2.0	2.1	2.0	1.0		1.0
Rapeseed meal		1.5		1.4		
Linseed			0.3			
Straw	0.5					
Wheat bran				1.5		
Supplements	3.4	0.2	0.6	0.5	9.5	2.5
Total	41.6	45.6	31.4	35.9	48.3	39.0
DMI	23.3	22.6	19.5	20.7	23.4	19.8
Starch, % DM	25.2	27.8	28.6	26.8	25.5	30.1
CP, % DM	14.7	14.0	14.8	13.2	13.1	13.2
EE, % DM	2.9	3.9	4.1	4.1	2.8	3.0
Ashes, % DM	6.6	6.7	7.5	7.4	7.8	6.4
NDF, % DM	38.7	37.4	35.4	36.9	38.5	38.8
MFU	20.0	20.7	17.9	17.9	21.3	17.5
PDIN, g/d	2.222	2.058	1.972	1.776	1.996	1.726
PDIE, g/d	2.154	2.043	1.940	1.784	1.960	1.785

(*F* farm, *DMI* dry matter intake, *DM* dry matter, *CP* crude protein, *EE* ether extract, *NDF* neutral detergent fiber, *MFU* milk fodder units, *PDIN* protein digested in small intestine when rumen-fermentable nitrogen is limiting, *PDIE* protein digested in small intestine when rumen-fermentable energy is limiting)

### Milk and blood sampling and assays

Milk was sampled on the day of the official record. Coccygeal vein blood samples were collected just before the morning milking and prior the feeding process. The same collection protocol was used across all farms. Blood was collected in PAXgene Blood RNA Tubes (PreAnalytiX GmbH, Switzerland), frozen 4 h after the collection and stored at -80 °C until the RNA isolation.

Prior to RNA isolation, blood samples were thawed at +4 °C for at least 12 h. RNA was isolated according to PAXgene Blood RNA Kit (PreAnalytiX GmbH, Switzerland) protocol.

Blood biochemical parameters, i.e., total protein, albumin, urea, glucose, creatinine, total bilirubin, cholesterol, AST/GOT (aspartate transaminase/glutamic oxaloacetic transaminase), gGT (gamma-glutamyl transpeptidase), zinc, ceruloplasmin, haptoglobin and paraoxonase were assayed as described in Sgorlon et al. [30]. Milk composition data, i.e., fat and total protein percentage, casein, urea, SCC and milk cortisol, were obtained from milk samples collected the same day of blood sampling and are described in Sgorlon et al. [31].

### Clustering of animals

To identify animals differing in their adaptive response to the environment, cows were clustered according to the level of acute phase proteins and molecules (total protein, albumin, zinc, ceruloplasmin, haptoglobin, paraoxonase, milk cortisol and SCC). To control the differences in adaptive response between breeds and to correct it for the potential effect of the environment the clustering was performed separately for each farm. For this the principal component analysis (PCA) using the correlation matrix in SPSS package was applied. According to the first two principal components the ten extreme individuals (five “plus” [APP+] and five “minus” [APP-] variants) from each farm were selected for gene expression analysis.

### RNA quality control and sequencing

First RNA was quantified and quality controlled by NanoDrop ND-1000 Spectrophotometer analysis (Thermo Fisher Scientific Inc., United States). Further RNAs with the highest quality was assigned the RNA integrity number (RIN) score by the Agilent 2100 Bioanalyzer (Agilent Technologies, United States) [32]. Finally samples with RIN ≥ 7 were selected for sequencing [33].

Previously was reported that high-throughput sequencing by RNA-Seq is highly reproducible within a large dynamic range of detection and provides an accurate estimation of RNA concentration in peripheral whole blood [20]. Thus, the experimental globin depletion from RNA samples was avoided as it could significantly reduce the amount and quality of isolated RNA and biological samples in our occasion were not possible to replenish.

The 60RNA samples (30 APP+ and 30 APP- variants) were sequenced by RNA-seq technology with the Illumina pipeline [24–27]. Reads obtained from the sequencing were aligned against *Bos taurus* UMD 3.1 reference genome assembly [34].

### Post-sequencing analysis

Raw counts produced by RNA-seq were normalized with the DeSeq2 software [27, 35]. To identify differentially expressed genes APP+ and APP- cows were compared either ignoring or considering their breed of origin: i) all APP+ vs. all APP-; ii) IH APP+ vs. IH APP-; iii) IS APP+ vs. IS APP-.

For each comparison, normalized RNA-seq data were analyzed with DeSeq2 software to calculate differential expression values (as log_2_ of the fold change) and raw *P*-values. To identify the significant genes raw *P*-values were corrected with the false discovery rate (FDR) method [36], using the cutoff of 0.05.

### Dynamic impact approach analysis

Gene expression data were also analyzed by the “dynamic impact approach” (DIA) developed by Bionaz and colleagues [28] for the transcriptome analysis.

DIA produces the list of the most impacted pathways integrating information coming from the dataset of the whole list of genes (regardless of their significance), differential expression values, FDR correction factor and raw p-value calculated by the DeSeq2 software. Graphically, the output is well demonstrated through two types of bars: the Impact bar indicating entity of the impact (colored in blue), and the Flux bar showing direction of the impact (red color represents the overexpression of the pathway, green color represents the under-expression of the pathway and yellow color indicates the absence in expression differences).

### Annotation clustering

Significant genes were submitted to the Database for Annotation, Visualization and Integrated Discovery (DAVID) to perform a serial annotation clustering [37]. This pipeline allowed us to form series of clusters with genes grouped according to their biological function. *P*-values automatically associated to each cluster were corrected by the Benjamini-Hochberg method. Clusters were considered significant if corrected *P*-values were lower than 0.05. Thereafter, significant genes from different clusters were grouped in a single list and further checked across comparisons to find out genes in common. The clustering procedure was applied separately for each comparison.

### Statistical analysis of milk production data

To investigate differences in milk composition across farms and APP groups, a mixed model analysis of variance (ANOVA) with nested design and Fisher’s least significant difference (LSD) test was applied for each breed separately. Data were analyzed with the SPSS package using the following statistical model:

$$ {Y}_{ijk}=m+ Far{m}_i+ APP{(Farm)}_{ji}+{e}_{ijk} $$

Where:

- *Y*_*ijk*_: dependent variable;
- *m*: general mean;
- *Farm*_*i*_: fixed effect for the farm, with *i* ranging from 1 to 3;
- *APP(Farm)*_*ji*_: nested effect for the APP group of animals, with *j* ranging from 1 to 2 within the *i* ^*th*^ farm;
- *e*_*ijk*_: residual error.

## Results

### Principal component analysis

**Fig. 1:**
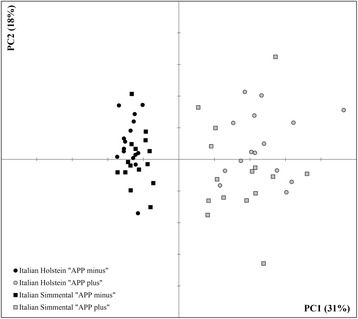
Clustering of IH and IS animals into APP+ and APP- variants. The graph summarizes six PCA done separately for each farm

The result of PCA is plotted in Fig. 1. The total variability explained by the first two components for each PCA separately was on average 50% (with a range from 43 to 54%) for the 6 commercial farms. The first component explained on average 31% of variability (range of 27-36%) and the second component explained on average 18% of variability (range of 15-25%). More accurate information about % of variability explained by PCA is reported in Table 4. Variables related to ceruloplasmin, haptoglobin and SCC were among the most important as they were highly correlated with the 1st PC within each farm (PCA). Their loadings correlation values ranged from 0.4-0.8 for each farm. Variable related to the total proteins was also very important as it was highly correlated (around 0.7) to the PC1 in the 4 out of 6 tested farms. Characteristics of groups of animals chosen for the final transcriptome analysis in the selected commercial farms are reported in Additional file 1.

**Table 4:** The total variability explained by the two first components for each PCA separately

% Explained variance						
	F1	F2	F3	F4	F5	F6
	IH	IH	IH	IS	IS	IS
PC1	27.5	33.9	27.2	29.1	35.4	35.6
PC2	19.2	18.0	15.8	24.6	16.7	14.6
Total	46.7	51.9	43.0	53.7	52.1	50.2

(*PC* principal component, *F* farm, *IH* Italian Holstein, *IS* Italian Simmental)

### Statistical analysis of milk production data

Significant differences between plus and minus APP groups were observed for milk urea in IS (*P* ≤ 0.001). Other parameters did not show significant differences (Table 5).

**Table 5:** Differences in milk parameters among commercial farms and APP groups of animals with *p*-values and mean standard errors of the statistical analysis performed with SPSS package

Italian Holstein								
Milk production data	F1	F2	F3	APP-	APP+	P_Farm_	P_APP(Farm)_	MSE, %
Milk yield, kg	39.5	37.3	33.0	40.7	32.6	0.231	0.086	4.90
Fat, %	3.08	3.99	3.52	3.37	3.70	*	0.351	2.98
Proteins, %	3.15	3.28	3.17	3.18	3.21	0.471	0.630	0.65
Casein, %	2.50	2.55	2.53	2.53	2.52	0.837	0.601	0.57
Urea, mg/dL	15.75	19.77	19.27	20.41	16.12	0.103	0.092	5.38
Italian Simmental								
Milk production data	F4	F5	F6	APP-	APP+	P_Farm_	P_APP(Farm)_	MSE, %
Milk yield, kg	22.4	33.8	28.3	29.5	26.7	**	0.219	4.43
Fat, %	3.80	3.35	3.27	3.34	3.61	0.208	0.503	3.94
Proteins, %	3.77	3.32	3.31	3.47	3.47	***	0.287	0.58
Casein, %	2.88	2.62	2.56	2.70	2.66	**	0.459	0.58
Urea, mg/dL	21.62	19.57	20.68	23.12	18.12	0.453	***	2.87

(*: low significance [*P* ≤ 0.05]; **: high significance [*P* ≤ 0.01]; ***: very high significance [*P* ≤ 0.001])

Between farms, IS cows showed significant differences in milk protein percentage (*P* ≤ 0.001), milk yield (*P* ≤ 0.01) and percentage of caseins (*P* ≤ 0.01). For IH animals the statistically significant differences between farms were observed only for milk fat percentage (*P* < 0.05).

Unlike IS animals, APP+ IH animals demonstrated a marked, even if not significant decrease in milk yield. Milk urea in APP+ animals showed a marked decrease in absolute values in both breeds; however in IS breed the decrease reached the significant level (*P* < 0.001).

### Post-sequencing analysis

**Fig. 2:**
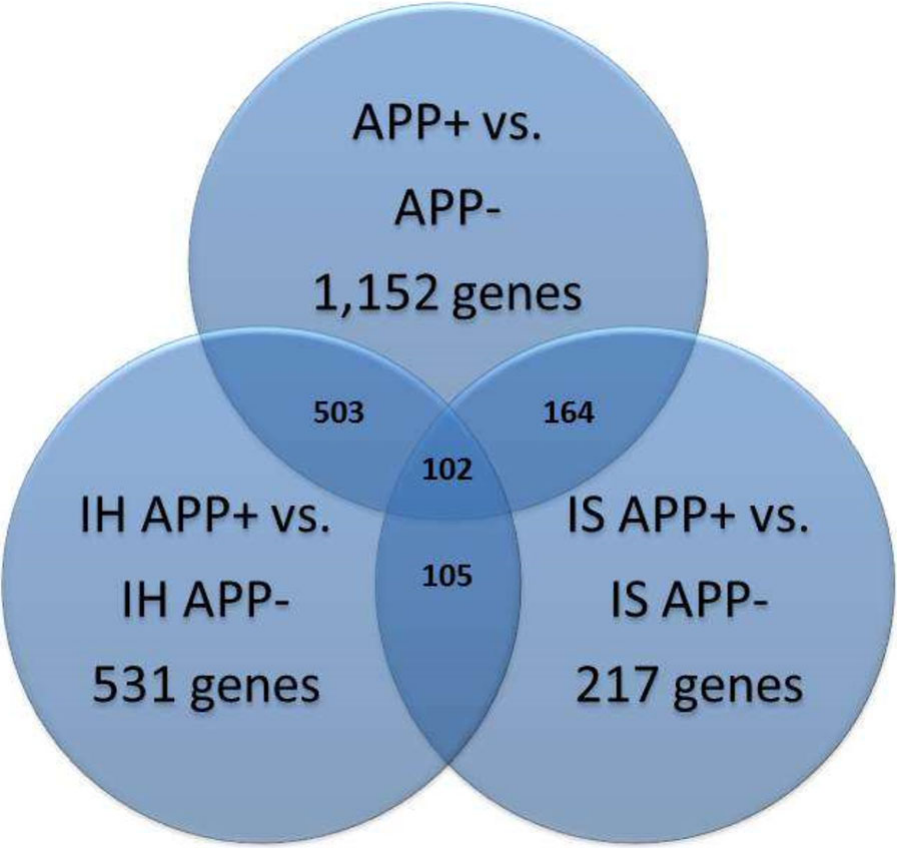
The number of shared and unique transcripts within each comparison

Alignment of RNA-seq data to the UMD 3.1 bovine reference genome identified 13,665 genes expressed in bovine blood cells. A total of 1,152 significant differentially expressed genes (*P* < 0.05) were identified in the comparison of all APP+ vs. all APP- animals across breeds; 531 in comparison of IH APP+ vs. APP- and 217 in comparison of IS APP+ vs. APP-. The number of shared and unique transcripts within each comparison is indicated on the Venn diagram (Fig. 2).

The higher number of significant genes obtained in the global comparison among APP+ and APP- cows may be explained by the procession of data from all sampled animals, hence each gene had expression data from both IF and IS cows. This fact increased the level of significance of number of genes in the comparison of all APP+ vs. all APP- variants and showed a less robust significance within intra-breed comparisons.

**Fig. 3:**
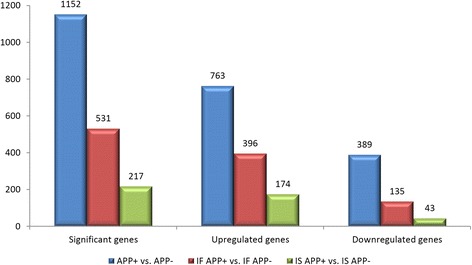
The number of upregulated and downregulated genes within each comparison

This analysis also allowed to evaluate the number of over- and underexpressed genes in the list of significant DE genes. Since the expression rate was indicated as the log_2_ of the fold change, we assumed that genes with an expression rate greater than 1 were overexpressed and those with the expression rate lower than 1 were underexpressed. The three comparisons, despite the great diversity in the number of significant genes, showed similar ratios between over- and underexpressed genes: in each case, the number of overexpressed genes was far greater than the number of underexpressed ones (Fig. 3). In the global comparison between APP+ and APP- cows the upregulated genes were about 2-fold higher than the downregulated: 763 overexpressed and 389 underexpressed genes. In IF comparison overexpressed genes were about 3-fold higher than underexpressed ones: 396 overexpressed and 135 underexpressed genes. In IS comparison overexpressed genes were about 4-fold more numerous than underexpressed ones: 174 overexpressed and 43 underexpressed genes.

These data highlights that the adaptive response affects blood transcriptome principally by increasing the expression of a high number of genes, while the downregulation is a mechanism with much lower extent.

### Dynamic impact approach

**Fig. 4:**
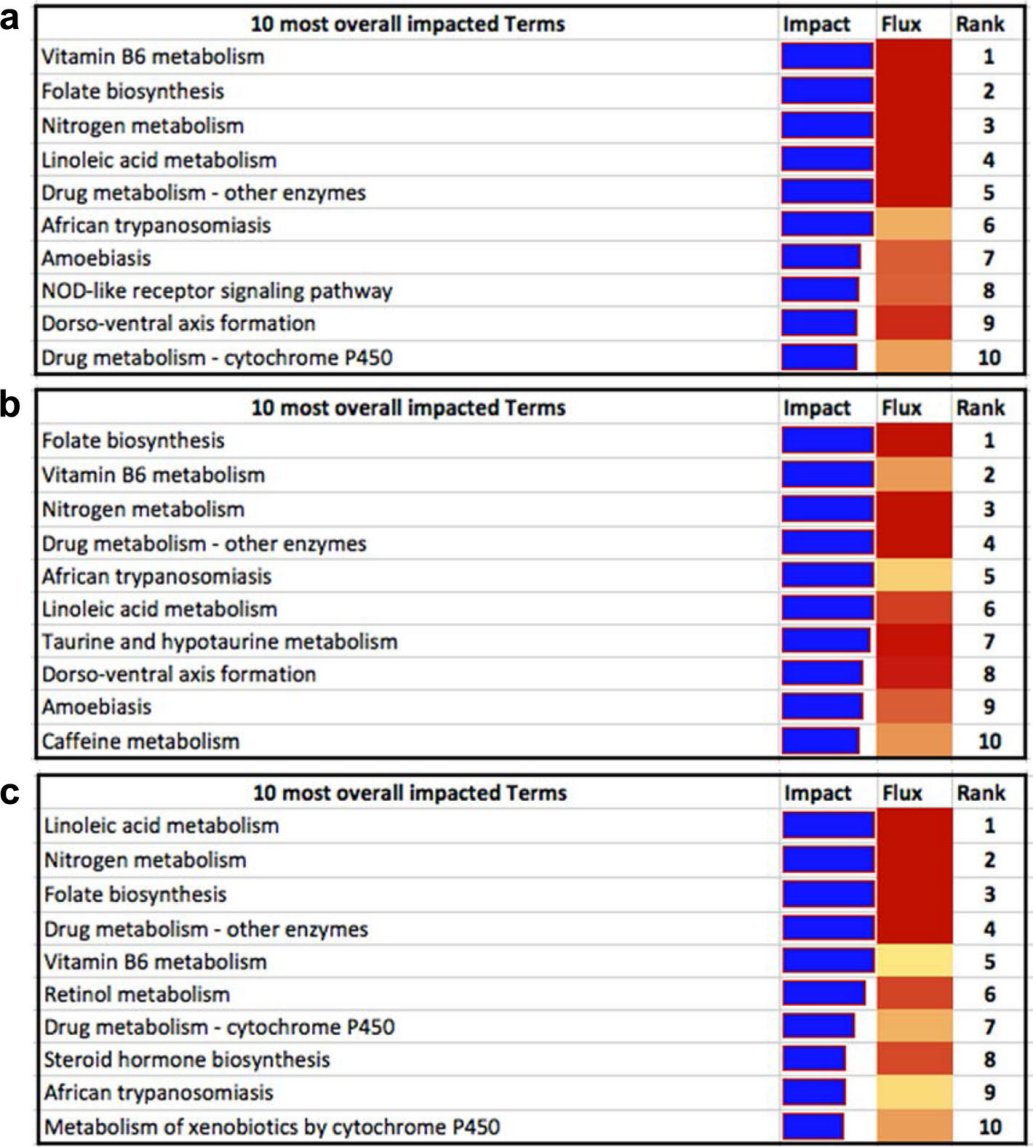
DIA outputs for each comparison (**a**: APP+ vs. APP-, **b**: IH APP+ vs. IH APP-, **c**: IS APP+ vs. IS APP-)

The ten most impacted KEGG pathways were identified by DIA for the comparisons with or without breed consideration (Fig. 4).

The three most impacted pathways in the comparison APP+ vs. APP- across breeds and within IH (Figs. 4a and b) are vitamin B6 metabolism, folate biosynthesis and nitrogen metabolism. In IS (Fig. 4c) these pathways are within the first five, in particular nitrogen metabolism is the second, folate biosynthesis the third and vitamin B6 metabolism the fifth most impacted pathway. Other pathways found in all three comparisons are linoleic acid metabolism, drug metabolism-other enzymes and African trypanosomiasis. Some other pathways are present in one comparison or in two out of three comparisons. Amoebiasis and dorso-ventral axis formation are present in the comparison across breeds and in IH; drug metabolism-cytochrome P450 is present in the comparison across breeds and in IS; NOD-like receptor signaling pathway is present only in the comparison across breeds; taurine and hypotaurine metabolism and caffeine metabolism pathways are present only in the IH comparison; retinol metabolism, steroid hormone biosynthesis and metabolism of xenobiotics by cytochrome P450 are present only in the IS comparison.

The complete list of all impacted pathways in each comparison is presented in Additional file 2.

### Gene annotation clusters

Function terms of each cluster identified by DAVID within each comparison were predicted by Gene ontology (GO) (Table 6). After elimination of repeated gene terms, 369 significant differentially expressed genes across breeds, 173 in IH and 73 in IS remained included in significant clusters.

**Table 6:** Groups of significant clusters in each comparison with indication of the Benjamini-Hochberg-corrected P-values (significance threshold: *P* < 0.05) and the number of genes in common among the three comparisons (see Table 7). Clusters were produced using DAVID database

Annotation Cluster	# Genes	Function terms	Corrected *P*-value	# Common genes
*APP+* vs. *APP-*				
C1	162	Purine nucleotide binding	0.001	2
C2	44	Cytoplasmic vesicle	0.003	1
C3	37	Cell fraction	0.009	2
C4	15	Positive regulation of cytokine production	0.009	N.A.
C5	150	Glycoprotein	0.016	23
C6	32	Response to wounding	0.018	1
C7	21	Regulation of cytokine production	0.026	1
C8	25	Leukocyte activation	0.029	N.A.
*IH APP+* vs. *IH APP-*				
CH1	11	Tyrosine protein kinase	0.003	N.A.
CH2	72	Purine nucleotide binding	0.005	2
CH3	21	Protein dimerization activity	0.006	N.A.
CH4	83	Glycoprotein	0.006	23
CH5	23	Cytoplasmic vesicle	0.035	1
*IS APP+* vs. *IS APP-*				
CS1	6	Calcium-binding region	0.004	N.A.
CS2	5	Anchored to membrane	0.016	3
CS3	7	Enzyme inhibitor activity	0.017	1
CS4	21	Extracellular space	0.018	12
CS5	5	Negative regulation of molecular function	0.018	N.A.
CS6	3	Cytokine biosynthetic process	0.019	1
CS7	27	Glycoprotein	0.024	19
CS8	42	Glycoprotein	0.025	23
CS9	10	Positive regulation of molecular function	0.030	4
CS10	15	Organelle lumen	0.033	1
CS11	4	Response to steroid hormone stimulus	0.034	2
CS12	5	Nuclear membrane	0.039	1

A total of 24 genes were differentially expressed in all 3 comparisons. Gene names and differential expression values for each of the 24 genes within each group are listed in Table 7.

**Table 7:** Common genes for the Annotation Clusters (see Table 6) and the relative differential expression values within each comparison

Gene name	Differential expression (n-fold)		
	APP+ vs. APP-	I﻿H APP+ vs. IH APP-	IS APP+ vs. IS APP-
*CA4*	3.32	3.68	3.00
*ALPL*	3.81	4.89	2.97
*IGF2*	0.33	0.26	0.42
*IL10*	1.67	1.90	1.47
*IGF2R*	2.50	1.91	1.36
*IL2RA*	2.18	2.23	2.14
*SCARB1*	1.67	1.97	1.42
*SLC6A2*	2.56	2.79	2.35
*MMP9*	1.84	2.03	1.66
*CHI3L1*	2.25	2.87	1.76
*PROK2*	2.27	2.42	2.13
*NMUR2*	2.76	2.92	2.60
*IL34*	0.50	0.46	0.54
*ACE2*	2.19	2.51	1.91
*HEPACAM2*	1.97	2.21	1.75
*A2M*	1.85	2.18	1.57
*TMEM120A*	1.54	1.83	1.29
*CD163*	2.61	3.85	1.77
*GPR84*	2.17	2.57	1.83
*PTX3*	5.50	6.71	4.51
*LYPD8*	0.44	0.46	0.43
*DEFB7*	1.94	2.00	1.88
*GNAL*	1.60	1.79	1.43
*DEFB10*	2.19	2.10	2.28

## Discussion

The aim of the present study was to investigate the impact of stress response on gene expression patterns in peripheral blood cells of lactating cows. The analysis of transcriptome variation was performed after the peak of lactation as the transition period is the most challenging in dairy cows and can interfere with the metabolic imbalance of animals [38]. Considering that animals on each commercial farm were kept under the same environmental conditions and that the farm factor was considered in the statistical model, the influence of management on animal adaptive response should have been minimized. Hence, the different levels of plasma APPs are likely to result from individual animal response to subclinical inflammatory/infective events or other stresses, since cows did not show visible clinical signs or symptoms of the presence of functional disorders.

Stress response is a very complex phenomenon as it can affect overall physiology through different mechanisms, like activation of sympathetic nervous system with the release of catecholamines, activation of HPA axis and non-circadian production of glucocorticoids [1, 4, 39], onset of an acute phase response [14]. Activation of these mechanisms may cause harmful and sometimes irreversible effects on many body systems. Stress may affect circulating glucocorticoids [4, 40, 41] with consequences on female reproductive system [42], immune system, osteoblastogenesis and bone metabolism [43–45], muscle production [46], metabolism of nutrients [47–49], functioning of the thyroid gland [50] and growth hormone axis [51]. Stress research is therefore complicated by these complex and diverse mechanisms and by individual and interspecies differences in stress response [17]. Understanding the biological basis of stress response in livestock is important for improving animal welfare in intensive production systems. In addition of being a growing public concern, animal welfare is important for production efficiency and influence both farm economy and environmental footprint. Whole-transcriptome analysis is crucial to understand how stress influences gene expression to elicit the complex phenomenon of adaptive response. Here we investigated differential expression in blood samples obtained from cows with high and low levels of positive APPs as proxy of stress status and identified stress response-related genes pathways in white blood cells.

### Impacted pathways by DIA

Once fed a list of differentially expressed genes, DIA exploits an online sheet of the Kyoto Encyclopedia of Genes and Genomes (KEGG) database [52] to detect significantly impacted pathways. It calculates the entity and direction of the impact and whether the pathway is entirely overexpressed, underexpressed or if the expression is not altered at all.

The most impacted pathways in APP+ cows in across and within breed analyses are presented in the Fig. 4. Significant genes in significant KEGG pathways have been analyzed in detail to understand gene and pathway function, since the names of KEGG pathways reported in the DIA output files are sometimes misleading.

The pathway of vitamin B6 metabolism (KEGG bta00750), was among the three most significant ones (rank 1 across breeds and in IH and rank 5 in IS). In this pathway we found two significant genes, *PDXK* (pyridoxal kinase) and *AOX1* (aldehyde oxidase 1). The latter was not significant in IS comparison, but it shows xa similar expression pattern. Pyridoxal kinase is involved in the ATP-dependent phosphorylation of pyridoxal, pyridoxamine and pyridoxine to pyridoxal-5-phosphate (PLP), pyridoxamine-5-phosphate and pyridoxine-5-phosphate, respectively. There is a requirement for ubiquitous expression of piridoxal kinase in mammalian tissues as PLP, before entering a cell, must be dephosphorylated and after diffusing through cell membranes it is converted back to the active cofactor by cytosolic pyridoxal kinase [53]. PLP is a very important enzymatic cofactor, as it participates to all transamination reactions and, in some cases, to decarboxylation, deamination and racemization of amino acids [54], catalyzes the rate-limiting step in glycogenolysis [55]. The impact of PLP on amino acid metabolism has direct consequences also on protein synthesis, whose physiological level is altered during an adaptive response. This evidence suggests that overexpression of *PDXK* gene is fundamental for metabolism regulation during adaptive phenomenon, as the cofactor affects protein and energy metabolism, that are likely increased during adaptation, and this is true not only for peripheral white blood cells, but for the vast majority of tissues in an organism. Aldehyde oxidase 1 is an enzyme mainly found in liver, shows broad substrate specificity, including pyridoxal, and catalyzes the oxidation of several endogenous and exogenous aldehydes, with the production of hydrogen peroxide and superoxide ion. Aldehyde oxidase isolated from polymorphonuclear leukocytes showed a more narrow substrate specificity: for example, the enzyme found in leukocytes is inactive on xanthine [56]. This enzyme has a role in oxidative stress and regulation of reactive oxygen species (ROS) homeostasis [57]. The catalysis requires the presence of flavin adenine dinucleotide (FAD) and a molybdopterin cofactor (MoCo) [57–59]. The enzyme also has a role in nitric oxide (NO) biosynthesis [60]. NO has various functions in the organism; white blood cells, mainly macrophages, secrete it as a chemical defense against bacteria and to induce vasodilation [61]. The involvement of aldehyde oxidase 1 in several oxidative metabolisms, in oxidative stress and in NO signaling may explain the overexpression of *AOX1* gene in APP+ cows.

Interesting significant genes emerged from the analysis of folate biosynthesis pathway (KEGG bta00790; rank 2 across breeds and in IH, rank 3 in IS). These are *ALPL* (alkaline phosphatase liver/bone/kidney) and *MOCS1* (molybdenum cofactor synthesis 1). The *ALPL* gene is mainly expressed in neutrophils and monocytes [62]. The role of alkaline phosphatase is fundamental as a high number of signal transduction cascades are involved in adaptive processes. Phosphorylation and dephosphorylation of signal proteins is the key determinant in the phenomenon that regulates the transduction and the amplification of a stimulus from the cell membrane receptor to the nucleus, where the modulation of gene expression occurs. Elevations in plasma alkaline phosphatase, whose sources include neutrophils and monocytes, can be also related to pathological conditions [62]. *MOCS1* encodes for a protein involved in the biological activation of molybdenum and it is highly expressed by peripheral white blood cells. Participating in the production of MoCo, it is indirectly involved in the catalytic activity of several enzymes, including aldehyde oxidase and xanthine oxidase [63]. *MOCS1* was significant only in APP+ vs. APP- comparison across breeds and the involvement of the gene in a number of metabolic oxidative pathways is likely the reason for its significant overexpression in the APP+ bovines.

In nitrogen metabolism pathway (KEGG bta00910; rank 3 across breed and in IH, rank 2 in IS), *GLUL* (glutamate-ammonia ligase) and *CA4* (carbonic anhydrase IV) genes were significantly differentially expressed. Glutamate-ammonia ligase is a PLP-dependent enzyme that produces glutamine from glutamate and free NH_3_. Glutamine is a common metabolite in many amino acid, purine and pyrimidine biosynthetic pathways, so this enzyme has a major role in protein and nucleic acid metabolism. It is also involved in acid-base homeostasis, cell signaling, cell proliferation and biosynthesis of γ-aminobutyric acid (GABA) [64]. Carbonic anhydrase IV is an important Zn-dependent enzyme present in several tissues and, among leukocytes, it is expressed principally by eosinophils and neutrophils. It has a main role in the control of acid-base balance in blood and other tissues [65]. Particularly, this isoform exists in the form of a glycophosphatidylinositol (GPI)-anchored protein and plays an important role in maintaining an appropriate cellular environment for the reactions that occur during adaptive responses. Both these genes are overexpressed in APP+ cows.

The pathway for linoleic acid metabolism (KEGG bta00591; rank 4 across breeds, rank 6 in IH, rank 1 in IS) includes a number of significant genes involved in inflammatory response and metabolism of drugs and xenobiotics. The phospholipase A2 genes, *PLA2G4F* and *PLA2G4A*, selectively hydrolyze membrane phospholipids. The first one has high selectivity for phosphatidylethanolamine, hydrolyzing the ester bond in sn-2 position, and has a role in mitogen-associated protein kinase (MAPK) and Ras signaling pathways [66]. The latter pathway leads to the production of free arachidonic acid, which is further converted in eicosanoids, involved in inflammatory response, and lysophospholipids, that are precursors of platelet-activating factor (PAF). Hence, this enzyme has a role in inflammatory response and hemodynamics, and is also involved in MAPK and G protein-coupled receptor (*GPCR*) signaling pathways [67]. Some significant genes in this pathway belong to cytochrome P450 superfamily (in detail: *CYP2E1*, *CYP3A4*, *CYP3A5*). These genes are also involved in metabolism of steroid hormones, drugs and carcinogens, playing a role in steroid-mediated physiological responses, activation and metabolic drug clearance and carcinogenesis [68–70].

In the pathway of drug-metabolizing enzymes (KEGG bta00983; rank 5 across breeds, rank 4 in IH and IS) there are several significant genes with a role in the metabolism of nucleotides, suggesting their role in an adaptive response. Xanthine dehydrogenase (*XDH*) is a paralog of *AOX1*, which can operate either as a dehydrogenase or as an oxidase. Xanthine dehydrogenase is involved in metabolism of hypoxanthine and xanthine and in the generation of ROS [71]. Recently, its role in recruiting macrophages through inflammasome activation has been investigated [72]. Cytidine deaminase (*CDA*) preserves pyrimidine pool by irreversibly deaminating cytidine and deoxycytidine to uridine and deoxyuridine, respectively. It is also involved in antibody diversification [73]. Uridine phosphorylase (*UPP1*) reversibly cleaves ribose-1-phosphate and deoxyribose-1-phosphate from uridine and deoxyuridine, releasing free uracil [74]. Another significant enzyme involved in pyrimidine metabolism is dihydropyrimidine dehydrogenase (*DPYD*) [75].

In African trypanosomiasis (KEGG bta05143; rank 6 across breeds, rank 5 in IH, rank 9 in IS) and amoebiasis (KEGG bta05146; rank 7 across breeds, rank 9 in IH) pathways we found a large number of significant genes directly involved in the onset of a stress response or inflammation. Among these, we found several proinflammatory cytokine genes, as *IL12B*, *IL18*, *IL1B* and *TNFα*. These cytokines are produced by different types of immune cells involved in growth, differentiation, chemotaxis and proliferation of white cells during an inflammatory event [76–80]. *NFkB1* and *RELA*, the nuclear transcription factor genes forming the same protein complex, were also among affected ones. *NFkB1* is activated by cytokines, free radicals, UV ray and pathogens’ products and it is involved in regulation of inflammation-mediated pathways [81] and in regulation of the expression of number of genes involved in cell adhesion and migration across vascular endothelium, like vascular cell adhesion molecule (*VCAM1*), laminin genes (*LAMA4*, *LAMC1*), fibronectin (*FN1*) and integrin genes (*ITGB2*, *ITGAM*). Some of the latter genes are strongly induced by cytokine signaling, so they have a primary importance in leukocyte adhesion and in cell signal transduction [82–86]. Significant genes in the African trypanosomiasis and amoebiasis pathways include also a number of cell surface receptors involved in inflammation signaling cascades, regulation of cell physiology during these events and in functionality of activated leukocytes. Among these genes there are Fas cell surface death receptor (*FAS*), toll-like receptors (*TLR2*, *TLR4*), *CD14* molecule and complement protein genes (*C8A*) [87–90]. These receptors transducer signals through different molecules, including myeloid differentiation factor (*MYD88*) [91]. Moreover, some significant genes in these pathways include enzymes that regulate the production of second messengers, like phosphatidylinositide-3-kinases (*PIK3R2*, *PIK3CD*) [92] and phospholipases C (*PLCB3*, *PLCB4*) [93], and proteases that regulate cell protein homeostasis, limiting tissue damage produced by overexpressed proteolytic enzymes, like serpin peptidases (*SERPINB1*, *SERPINB3*) [94]. In IS breed amoebiasis pathway was found to be not significantly impacted between APP+ and APP- animals.

NOD-like receptor signaling pathway (KEGG bta04621) was found to be significant only in the APP+ vs. APP- across breed comparison (rank 8). Significant genes belonging to the pathway are mostly involved in regulation of cell cycle and pro-apoptotic signaling, interacting with the nuclear factor NFkB1 to activate it. Pro-apoptotic genes include the nucleotide-binding oligomerization domain containing receptors (*NOD1*, *NOD2*) [95] and genes of the caspase recruitment domain family (*CARD6*, *PYCARD*, *NLRP3*), which also participate to the formation of inflammasomes [96–98]. In this pathway there are also two significantly overexpressed molecular chaperones, *HSP90AA1* and *HSP90B1*.

Dorso-ventral axis formation (KEGG bta04320) pathway was significantly impacted in APP+ vs. APP- across breeds (rank 9) and in IH (rank 8). However, some genes belonging to this pathway were significant also in IS breed. In this pathway there are two significant transcription factors, *ETV6* and *ETS2*, which are oncogenes with a role in hematopoiesis and apoptosis [99, 100].

The cytochrome P450-mediated metabolism of drugs (KEGG bta00982) is among the significantly impacted pathways across breeds (rank 10) and in IS (rank 7). Important genes involved in oxidative stress and detoxification of oxidation by-products are included in this pathway, such as membrane-bound microsomal glutathione S-transferase (*MGST1*), with a role in the development of inflammation and in cellular defense [101], aldehyde dehydrogenase (*ALDH3B1*) [102] and monoamine oxidase A (*MAOA*) [103]. Monoamine oxidase A has a role in the metabolism of serotonin: this molecule has been shown to be synthesized, released and degraded also by T lymphocytes [104]). Among the significant genes, we found also some terms significantly overexpressed in other pathways, as *AOX1* and *CYP2E1*.

Metabolism of taurine and hypotaurine (KEGG bta00430) was strongly impacted only in IH (rank 7) and the only significant gene detected was a member of γ-glutamyltransferase family (*GGT5*). This gene is involved in metabolism of glutathione and leukotrienes and plays a role in oxidative stress and inflammatory response [105].

The pathway of caffeine metabolism (KEGG bta00232) showed a high impact only in IH (rank 10). It includes the gene *XDH*, the function of which has been previously discussed.

Retinol metabolism (KEGG bta00830) was highly impacted only in IS (rank 6). Important differentially expressed genes in this pathway are *AOX1*, *CYP3A4* and *CYP3A5* and their role in adaptive response was described previously.

Another pathway impacted only in IS included steroid hormone biosynthesis pathway (KEGG bta00140; rank 8). It included *CYP3A4*, *CYP3A5* and *CYP2E1* genes, involved in the biosynthesis of different types of steroids, including corticosteroids, which have a relevant role in adaptive response.

The same scenario occurred also in the pathway relative to metabolism of xenobiotics mediated by cytochrome P450 (KEGG bta00980; rank 10 in IS). It included genes *MGST1*, *CYP2E1* and *ALDH3B1*, which are significant also in other pathways.

The same significant gene plays different roles in different pathways, for example in the metabolism of different compounds or in the signaling of a number of signal transduction cascades. Thus, to obtain a comprehensive analysis and to confirm the significance of the relevant genes, the most impacted pathways were explored by DAVID.

### DAVID annotation clusters

Annotation clusters produced by DAVID online tool confirmed the significance of a large number of genes involved in adaptive response. Genes were clustered according to their common structural characteristics or molecular function. Table 6 summarizes the significant clusters, the number of total genes per cluster and the number of genes in common with at least another cluster. In Table 7 are listed common genes between all three comparisons. A comprehensive list of genes included in each cluster is available in Additional file 3.

The highest number of common genes among clusters was included in C5, CH4 and CS8, which are clusters of glycoproteins, based on the GO terms. Twenty-three out of 24 shared genes are included in these clusters. The only one excluded is the *GNAL* gene. We can assume that genes present in these clusters are grouped only according to structural features, as they have various molecular functions, but are all classified as glycoproteins. *GNAL* is included in the cluster that reports purine nucleotide-interacting proteins.

In these clusters we can find some genes that were significant also in DIA output, i.e., *CA4*, *ALPL* and *IL10*. Other shared genes were not included in the most impacted pathways, but they have an important role in the regulation of adaptive response. For example, the receptor of insulin-like growing factor II (*IGF2R*) is overexpressed in all three comparisons, possibly because of its role in intracellular trafficking of lysosomal enzymes and degradation of IGF-II [106].

Angiotensin I-converting enzyme 2 (*ACE2*) has a direct effect on cardiac and renal functions [107]. Its overexpression may be another consequence of hyperactivation of HPA axis, as glucocorticoids have a direct effect on blood pressure and cardiovascular system, and of an increased uremia, as this condition may trigger the recruitment of pro-atherogenic, ACE2-expressing monocytes [108].

As most of cytokines, present in all three comparisons, interleukin 10 (*IL10*) is significantly overexpressed. Such overexpression can be explained by its important role in lymphocytes differentiation and proliferation and in the production of antibodies by B cells. In the same list of overexpressed genes are present receptors for cytokines, like interleukin 2 receptor alpha (*IL2RA*), involved in the intracellular signaling pathways.

The only one underexpressed cytokine revealed among three comparisons is the pro inflammatory cytokine IL34.

Defensins are small antimicrobial, cytotoxic peptides produced by neutrophils [109]. β-defensins (*DEFB7* and *DEFB10* in Table 7), belonging to one of the three existing groups of defensins, were overexpressed in all comparisons. Their overexpression could be associated to the presence of a bacterial infection in mammary gland [110] and this result is in agreement with the highly impacted pathway relative to *S. aureus* infection (see Additional file 2).

Another interesting overexpressed gene is the gene encoding matrix metalloproteinase 9 (*MMP9*). Its overexpression can be associated with an augmented production of hematopoietic stem cells [111] that give rise to all red and white blood cells and platelets. *MMP9* has recently been investigated for its role in promoting the secretion of pro-inflammatory cytokines and the migration of T cells towards inflammation sites. Moreover, the protein increases the permeability of brain-blood barrier to cytokines, showing an important involvement in neuroinflammation [112].

Summarizing the results, a lot of highly impacted pathways were found in common between IH and IS breeds, indicating a similar variation in the gene expression under environmental adaptation. This observation was also confirmed by annotation clustering of the significantly expressed genes: in fact, some of the significantly clustered genes are present also in the most impacted pathways, with similar levels of expression.

Differences between breeds were observed only on the level of individual genes. Therefore, considering the overall variation in stress-inducing factors and stress-related gene expression, the general patterns can be considered very similar in the two breeds investigated.

The obtained data can be considered reliable even if the further validation analysis of the differential gene expression in the population could improve the relevance of the conclusions. This point can be developed in a future research.

### Analysis of milk production data

The differences in milk production data between APP- and APP+ cows are reported in Table 5. Significant association with high APP level was observed only for milk urea in IS, which showed a marked decrease in APP+ animals. A marked decrease, close to being statistically significant, was observed also in IH milk yield and milk urea.

The decrease in milk yield can be associated to the onset of a stress response as energy resources are driven towards other organs and other more important physiological processes to guarantee the animal’s survival. The decrease in milk urea can be linked to the overall increased protein synthesis. In dairy cows milk urea reflects the catabolism of protein by the ruminant tissues and within the rumen by bacteria. The decrease of rumen ammonia may indicate that animals increase the protein synthesis during an adaptive response [113]. Indeed, high levels of APPs and at the same time high expression of gene products of several pathways were observed in APP+ animals.

## Conclusions

This transcriptomic study in lactating dairy cows from IH and IS breeds allowed to assess that the onset of an adaptive response to the environment involves a large number of pathways that are regulated in a similar way in the two breeds, with marginal differences in significantly over- or underexpressed single genes. The altered expression of these genes is statistically associated with the variations of only certain milk production parameters between groups of cows with activated and not activated stress response mechanisms.

Considering all the results we can conclude that the two studied breeds have similar patterns but vary in the degree of activation of metabolic and physiological mechanisms of adaptation to the environment. IH showed a higher rate of significant genes and impacted pathways, demonstrating a higher metabolic activity, respect to the IS breed.

## Additional files

Additional file 1: Characteristics of animals chosen for the transcriptome analysis in the selected commercial farms. F: farm, IH: Italian Holstein, IS: Italian Simmental, DIM: days in milk, BCS: body condition score, SCC: somatic cell count, values are expressed as mean ± SD. (XLSX 11 kb)
Additional file 2: Complete set of 10% most impacted pathways produced by DIA analysis in each comparison. Three images are presented. The first image (named A) collects the 10% most impacted pathways for APP+ vs. APP- comparison. The second image (named B) lists the 10% most impacted pathways for IH APP+ vs. IH APP- comparison. The third image (named C) lists the 10% most impacted pathways for IS APP+ vs. IS APP- comparison. (DOCX 211 kb)
Additional file 3: Complete list of genes included in DAVID Annotation Clusters. Each sheet of the.xlsx file includes one of the Annotation Clusters listed in Table 6 of the present article. In each Annotation Cluster the included genes are listed. The genes found to be common in each comparison are bolded. (XLSX 57 kb)

## Abbreviations

AP: Acute phase reaction
APP: Acute phase protein
DIA: Dynamic impact approach
F1: Farm 1
F2: Farm 2
F3: Farm 3
F4: Farm 4
F5: Farm 5
F6: Farm 6
HPA: Hypothalamic-pituitary-adrenal
IH: Italian Holstein
IS: Italian simmental
PCA: Principal component analysis
RIN: RNA integrity number
SCC: Somatic cell count
