# Supplementary figures and images for: Breed and adaptive response modulate bovine peripheral blood cells’ transcriptome
Source: J Anim Sci Biotechnol. 2017 Jan 25;8:11. doi: 10.1186/s40104-017-0143-y (PMC5264304; doi:10.1186/s40104-017-0143-y)

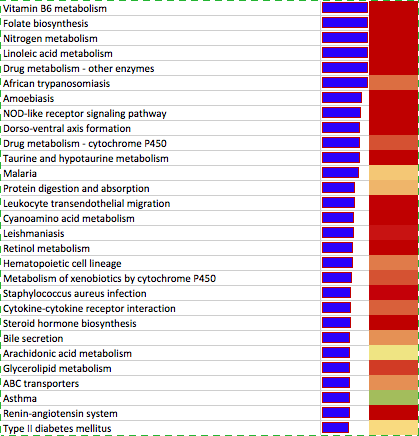


**A**


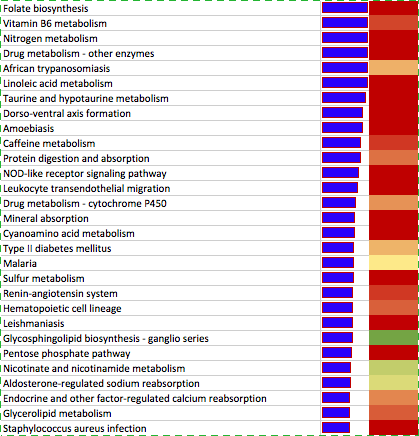


**B**


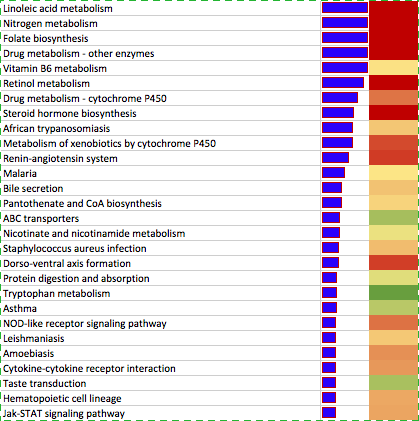


**C**

Supplement: Additional file 2: — Complete set of 10% most impacted pathways produced by DIA analysis in each comparison. Three images are presented. The first image (named A) collects the 10% most impacted pathways for APP+ vs. APP- comparison. The second image (named B) lists the 10% most impacted pathways for IH APP+ vs. IH APP- comparison. The third image (named C) lists the 10% most impacted pathways for IS APP+ vs. IS APP- comparison. (DOCX 211 kb) [file 40104_2017_143_MOESM2_ESM.docx]
